# Phages as antimicrobials against multi-drug resistant bacteria

**DOI:** 10.3389/fmicb.2026.1747240

**Published:** 2026-02-23

**Authors:** Salomé Plat, Gisèle LaPointe, Lawrence Goodridge

**Affiliations:** Department of Food Science, Canadian Research Institute for Food Safety, University of Guelph, Guelph, ON, Canada

**Keywords:** antimicrobials, bacteriophages, depolymerases, endolysins, multi-drug resistant bacteria, phage therapy

## Abstract

Multi-drug resistant bacteria (MDR) pose a major public health challenge. Their ability to exchange resistance genes through Horizontal Gene Transfer (HGT) promotes the appearance of resistant strains, limiting antibiotic treatments for infections caused by these MDR bacteria. Among alternative approaches, phage therapy stands out as a promising strategy that utilizes bacteriophages to specifically target and effectively eliminate bacteria. This narrative review provides an overview of the current knowledge on the use of whole bacteriophages as antimicrobial agents in human and veterinary medicine, as well as in the food industry whether used alone, in cocktails, or combined with antimicrobials. While whole phages offer high specificity and an efficient elimination of bacteria, their application is associated with several limitations, including their contribution to HGT, the emergence of bacterial resistance, their narrow host range, the immune recognition, and the difficulties posed by their regulation. To address these challenges, this review focuses on phage-derived enzymatically active proteins, such as endolysins and depolymerases, as alternative antimicrobial tools, used alone or in combination. These phage components, being smaller and structurally simpler than whole phages, behave more similarly to conventional antimicrobial compounds. They have so far presented a low risk of bacterial resistance appearance and less chance of immune response. In addition, their classification as antimicrobial enzymes or conventional biologics could facilitate regulatory approval by aligning with existing regulatory frameworks. A total of 40 studies were included in this narrative review, highlighting the outcomes of applications involving whole bacteriophages (*n* = 11) and phage-derived enzymes, including endolysins and depolymerases (*n* = 27).

## 1 Introduction

In animals and throughout the food chain, antibiotics have been used for years for prophylaxis, as growth promoters, or to treat infections, which exert constant selective pressure on pathogens, favoring the emergence of resistance. In response to the threat of antibiotic resistance, several countries and regions have introduced regulations restricting the use of medically important antibiotics in animal agriculture, particularly for non-therapeutic purposes such as growth promotion and routine prophylaxis. For example, since December 1, 2018, Canada has prohibited the use of Category I, II, and III antibiotics for prophylactic purposes or as growth promoters in animal production. These medically important antimicrobials may now only be used for therapeutic purposes and strictly under veterinary prescription, in accordance with the measures of Health Canada and the Canadian General Standards Board (Canadian Animal Health Institute, 2017; Government of Canada, 2021). In the United States, the FDA implemented directive GFI #213 in January 2017, which reclassified medically important antibiotics administered through feed or drinking water as prescription-only products or subject to a Veterinary Feed Directive (VFD). Since then, their use has been restricted to therapeutic purposes only, and any non-therapeutic use (including for growth promotion or routine prophylaxis) is prohibited. Large-scale administration through feed or water is only permitted under veterinary supervision and in strict compliance with the conditions of use defined by manufacturers (FDA, 2017). In Europe, the use of antibiotics as growth promoters in animal feed has been banned since January 1, 2006 (Commission européenne, 2005). Since July 2022, veterinary prescription has been mandatory for all antimicrobials, and their prophylactic use is now strictly limited to exceptional situations, involving either an individual animal or a small group, only when the risk of infection is very high and the potential consequences are severe (Agence Nationale de la Sécurité Sanitaire, 2022).

Antibiotic resistance is already widespread in the environment, leading to a loss of effectiveness of traditional antibiotics and leaving only a limited range of drugs for the treatment of bacterial infections. One of the most widely suggested alternatives to antibiotic treatments is phage therapy. Bacteriophages, also called phages, are viruses that infect and kill bacteria with a great specificity, and are among the most diverse and abundant microbes on our planet. Their presence was first described and observed in 1915 by Frederick William Twort, and in 1917 Félix d'Hérelle was the first to isolate and characterize phages (Twort, 1915; D'Hérelle, 1917). He popularized the term “bacteriophages” as well as their use in the treatment of bacterial infections. However, with the discovery of antibiotics in 1928 by Fleming (1929) and the introduction of penicillin in 1942, and given the easier use of antibiotics, many researchers shifted their attention, leading to the progressive decline of bacteriophage research and application in Western countries, until it completely disappeared in France in 1990 with the closure of the last two Pasteur Institutes (Paris and Lyon) that still had therapeutic bacteriophages (Gordillo Altamirano and Barr, 2019; Patey et al., 2019). At that time, the lack of sufficiently advanced tools to visualize phages and understand their mechanisms, chemical molecules such as antibiotics appeared to be the ideal candidates, sidelining bacteriophage studies—especially in a wartime context, where an immediate and operational therapeutic solution was required to treat infections. Given the alarming spread of antibiotic resistance, which increases the number of human deaths caused by multi-drug resistant (MDR) bacteria that cannot be treated with currently available antibiotics, along with the slowdown in the production of new antibiotic molecules—very costly and very slow—there has been a renewed interest in phage therapy since 2000 (Gordillo Altamirano and Barr, 2019; Saha and Mukherjee, 2019). Phage therapy can be used as a last resort against infections caused by MDR bacteria after the failure of standard treatments (Genevière et al., 2021; Melo et al., 2020; Patey et al., 2019).

### 1.1 Bacteriophage life cycle: lytic and lysogenic

Bacteriophages cannot multiply on their own, as they do not possess an autonomous replication system. They use the bacterial machinery to replicate and kill bacterial cells, depending on their life cycle. Their potential to act as antibiotics lies in their specificity to kill bacteria without infecting or affecting eukaryotic cells. There are two replication cycles of phages in the bacterial host cell: the lytic cycle and the lysogenic cycle (Cui et al., 2024; Harada et al., 2018). These two cycles begin in the same way: first, the attachment of the phage to the bacterium, which is mediated by receptor-binding proteins (RBPs) located on the surface of the phage, specific to the bacterial receptors on their surface (Cui et al., 2024). There are as many RBPs as there are bacterial receptors, which can be of different nature: lipopolysaccharides (LPSs), membrane proteins such as porins or transporters, teichoic acids and lipoteichoic acids, capsular polysaccharides, or pili and fimbriae (Bertozzi Silva et al., 2016). After attachment, the phages introduce their genome into the infected cell. It is the following steps that differentiate a lytic cycle from a lysogenic cycle.

The lytic cycle is characteristic of virulent bacteriophages, whose primary objective is to infect and ultimately lyse the host bacterium, thereby releasing numerous progeny phages (Cui et al., 2024; Harada et al., 2018; Pattnaik et al., 2024; Salmond and Fineran, 2015). Upon infection, the phage attaches to the bacterial surface and injects its genome into the host cell. Once inside, the viral genome hijacks the host's transcriptional and translational machinery to express phage-specific genes (Harada et al., 2018; Salmond and Fineran, 2015). These genes encode essential structural components of the virus, including capsomers (which self-assemble to form the protective capsid), tail fibers and baseplate proteins (involved in host recognition and attachment), and assembly proteins that orchestrate the construction of complete viral particles, known as virions (Harada et al., 2018; Pattnaik et al., 2024). During this phase, dozens or even hundreds of new phage particles are assembled within the bacterial cytoplasm. The final stage of the lytic cycle involves the synthesis of lysis proteins – notably endolysins, holins, and spanins (Harada et al., 2018; Pattnaik et al., 2024; Salmond and Fineran, 2015). These proteins work in concert to degrade the bacterial cell wall and membrane, causing the bacterium to rupture and release the newly formed virions into the surrounding environment. Because virulent phages rely exclusively on this destructive cycle to propagate, they are incapable of establishing long-term associations with their hosts (Salmond and Fineran, 2015).

In contrast, the lysogenic cycle is utilized by temperate (or non-virulent) phages, which adopt a more passive strategy (Harada et al., 2018; Salmond and Fineran, 2015). Instead of immediately destroying the host cell, temperate phages integrate their genomes into the bacterial chromosome, forming a dormant genetic element known as a prophage (Harada et al., 2018). In this state, the phage genome is replicated alongside the host DNA during cell division, enabling vertical transmission to daughter cells without producing new virions (Salmond and Fineran, 2015). The establishment of lysogeny begins with the injection of the phage DNA into the host bacterium. The linear phage genome circularizes—often due to complementary cohesive ends—and undergoes site-specific recombination with the bacterial chromosome (Salmond and Fineran, 2015). This recombination is mediated by two key proteins: the integrase (Int), which is encoded by the phage and facilitates DNA cleavage and ligation; and the Integration Host Factor (IHF), a bacterial protein that assists in aligning the DNA sequences. These enzymes target specific attachment sites: *attP* on the phage genome and *attB* on the bacterial chromosome. The successful recombination of these sites creates *attL* and *attR*, which flank the integrated prophage (Grindley et al., 2006). While in the lysogenic state, the prophage remains transcriptionally silent—its genes are not expressed, and no new phage particles are produced. However, this relationship is not permanent.

### 1.2 Prophage induction: environmental triggers and the switch to the lytic cycle

External stressors or environmental signals—such as ultraviolet (UV) radiation, DNA-damaging agents (e.g., mitomycin C), oxidative stress, or nutrient deprivation—can trigger the prophage to exit the lysogenic state in a process known as induction (Harada et al., 2018; Salmond and Fineran, 2015). This response is typically mediated through the bacterial SOS response, a global regulatory system activated by DNA damage. Under such conditions, the prophage excises itself from the bacterial chromosome, reversing the site-specific integration (Grindley et al., 2006). This excision process is catalyzed by the phage-encoded excisionase (Xis) in cooperation with integrase (Grindley et al., 2006). Once excised, the phage genome re-enters the lytic cycle: phage genes are expressed, new virions are assembled, and ultimately the host cell is lysed to release the progeny (Harada et al., 2018; Salmond and Fineran, 2015).

Thus, temperate phages possess the remarkable ability to alternate between a latent lysogenic state and a destructive lytic phase, depending on the environmental context. This dual lifestyle allows them to persist across generations while retaining the capacity for rapid propagation when conditions become unfavorable.

## 2 Review methodology

Studies included in this review were retrieved from the PubMed (NCBI, https://pubmed.ncbi.nlm.nih.gov) and Web of Science (Clarivate, https://www.webofscience.com) databases using the following keywords: *bacteriophages, phages, phage therapy, phage-based treatment, phage-enzymes, endolysins, depolymerases*, and *multidrug-resistant (MDR) bacteria*. The search focused on applications in *human medicine, veterinary medicine*, and *food*, while studies primarily addressing antimicrobial resistance (AMR) were excluded. Year of publication was restricted to between 2015 to March 2025.

The Covidence environment (https://www.covidence.org) was used to remove duplicates (by Covidence and manually), articles without open access to the full text, and publications in languages other than English or French. An initial screening was performed based on article titles, followed by a second screening based on abstracts. Abstracts that did not report the use of whole bacteriophages or phage-derived proteins as antimicrobial agents in human, veterinary, or food were excluded.

Full texts of the remaining articles were then reviewed, and the most comprehensive and relevant studies were selected for inclusion in this narrative review.

## 3 Whole phages as antimicrobials

The use of bacteriophages as antimicrobial agents spans multiple fields, from medicine to biotechnology and food safety, reflecting their versatility and growing importance in combating bacterial infections.

Historically, the therapeutic potential of phages was first recognized in 1919, when Félix d'Hérelle, a French-Canadian microbiologist, successfully used bacteriophages to treat dysentery in a human patient. This marked the beginning of phage therapy. D'Hérelle, along with Frederick Twort, a British bacteriologist who independently observed bacteriophages in 1915, is credited with the co-discovery of bacteriophages. In the early 1920s, d'Hérelle expanded phage therapy to treat a range of bacterial diseases, including cholera, bubonic plague, conjunctivitis, and various skin infections. The application of phages extended to animal models by the early 1980s. In 1983, researchers began using phages experimentally to treat infections such as septicemia and meningitis in animals, providing valuable preclinical data that demonstrated both efficacy and safety.

In the 21st century, the utility of bacteriophages broadened further into food safety. A major milestone occurred in 2006 with the commercial development of the first phage-based product specifically designed to eliminate *Listeria monocytogenes*—a dangerous foodborne pathogen. This innovation paved the way for the integration of phage-based biocontrol into food production and storage systems (Gordillo Altamirano and Barr, 2019).

Beyond therapeutic and food applications, bacteriophages have also been harnessed in biotechnology for a wide range of purposes. These include their use in the design of phage display systems for vaccine development, biosensors for the rapid detection of pathogenic bacteria, and surface disinfection technologies to control microbial contamination in clinical and industrial settings (Harada et al., 2018). Collectively, these applications demonstrate the versatility and potential of phages as precision tools for managing bacterial threats in a world facing problems treating outbreaks caused by MDR bacteria.

### 3.1 Human applications

In the medical field, several clinical trials have demonstrated the effectiveness of phages as curative treatments against MDR bacteria, notably those caused by members of the ESKAPE group (*Enterococcus faecium, Staphylococcus aureus, Klebsiella pneumoniae, Acinetobacter baumannii, Pseudomonas aeruginosa*, and *Enterobacter* spp.), which are responsible for hard-to-treat nosocomial infections (Miller and Arias, 2024). Phages have been administered intravenously, topically, or locally (inhalation, wound application, or instillation), in cases of pulmonary, bone and joint, or urinary infections, with positive clinical outcomes, including cases of complete remission (Melo et al., 2020; Patey et al., 2019) (Supplementary Material Table 1). Clinical trials have been conducted with encouraging results, including cases of complete remission, particularly in chronic bone and joint or pulmonary infections, but often in compassionate and individualized contexts, highlighting the lack of standardization (Melo et al., 2020). This lack of standardization is attributed to the high specificity of bacteriophages for a given strain, and therefore the impossibility of developing therapies applicable to all patients (Kortright et al., 2019). Other clinical trials have not shown convincing success of phage therapy, notably with the PhagoBurn trial (2015–2017) where researchers evaluated the effectiveness of a topical phage cocktail against *P. aeruginosa* in burn patients, which had to be prematurely terminated due to the lack of phage efficacy compared to the standard treatment (1% silver sulfadiazine cream) (Jault et al., 2019) (Supplementary Material Table 1). There are also cases of phage therapy failure showing a lack of efficacy of orally administered phages in the treatment of *Escherichia coli* and *Proteus* for diarrhea in children, or a phage treatment success rate similar to that of placebo in urinary tract infections (*Staphylococcus, Streptococcus, E. coli, P. aeruginosa*, and *Proteus*), reported by the authors as the cause of low phage titers, resulting in insufficient phage coverage below the phage replication threshold (Jault et al., 2019; Leitner et al., 2021; Sarker et al., 2016) (Supplementary Material Table 1).

### 3.2 Veterinary applications

In veterinary medicine, phage cocktails have been used both prophylactically and curatively, particularly to prevent or treat intestinal or respiratory infections in livestock animals, especially poultry, pigs, and cattle (Johnson et al., 2008).

In poultry, phages have been used to eradicate *Salmonella* spp., *Campylobacter jejuni, E. coli*, and *Clostridium perfringens*, major pathogens for both animal health and human food safety (Abd-El Wahab et al., 2023). Oral administration of phages via drinking water or feed significantly reduced intestinal colonization by *S. enterica, C. jejuni*, and *C. perfringens* in chickens, thus decreasing the risk of contamination in the food chain (Abd-El Wahab et al., 2023; Choi et al., 2024; Gigante and Atterbury, 2019; Johnson et al., 2008; Mosimann et al., 2021) (Supplementary Material Table 1). However, the reduction of *E. coli* was only significant by injection or inhalation of bacteriophages (Abd-El Wahab et al., 2023; Johnson et al., 2008).

In pigs, phage therapy has been studied for the control of *S. enterica* and *E. coli* (ETEC) infections, responsible for salmonellosis and diarrhea, respectively. Trials have shown that administration of phages alone or as phage cocktails can significantly reduce bacterial load (Choi et al., 2024; Ferriol-González and Domingo-Calap, 2021; Johnson et al., 2008) (Supplementary Material Table 1).

In cattle, phage therapy has been used to treat and prevent infections caused by *E. coli* O157:H7 and *S. aureus*. The objective here is not only therapeutic but also to reduce the prevalence of these pathogens on farms, thus limiting the risk of contamination of animal-derived products. Therapeutic trials using phages alone or in phage cocktails have also led to a significant reduction in colonization and bacterial growth, thereby reducing mortality (Choi et al., 2024; Johnson et al., 2008).

### 3.3 Food applications

In the agri-food sector, phages are used for bio preservation, to improve the microbiological safety of foods. They target and eliminate pathogens such as *Bacillus* spp., *E. coli* O157:H7, *L. monocytogenes, Salmonella* spp., *S. aureus, Shigella* spp., *Cronobacter sakazakii*, or *C. jejuni* on the surface of food products such as meat, dairy products, eggs, milk, poultry meat, seafood, or fresh fruits and vegetables (Dhulipalla et al., 2025; Fokas et al., 2025; Vikram et al., 2021) (Supplementary Material Table 1). Since 2006, commercial phage-based preparations such as EcoShield™, ListShield™, SalmoFresh™, ShigaShield™, and many others have been developed and approved for food applications to combat *E. coli* O157:H7, *L. monocytogenes, Salmonella* spp., and *Shigella* spp. (Dhulipalla et al., 2025; Gordillo Altamirano and Barr, 2019; Saha and Mukherjee, 2019; Vikram et al., 2021). Furthermore, innovative strategies have explored the incorporation of bacteriophages into food packaging materials, enabling a sustained antimicrobial activity that contributes to extended shelf life, reduced oxidation and microbial growth, and enhanced consumer protection against potential foodborne pathogens (Dhulipalla et al., 2025; López de Dicastillo et al., 2021; Wagh et al., 2023) (Supplementary Material Table 1).

### 3.4 Advantages and disadvantages of whole phages as antimicrobials

**Figure 1:**
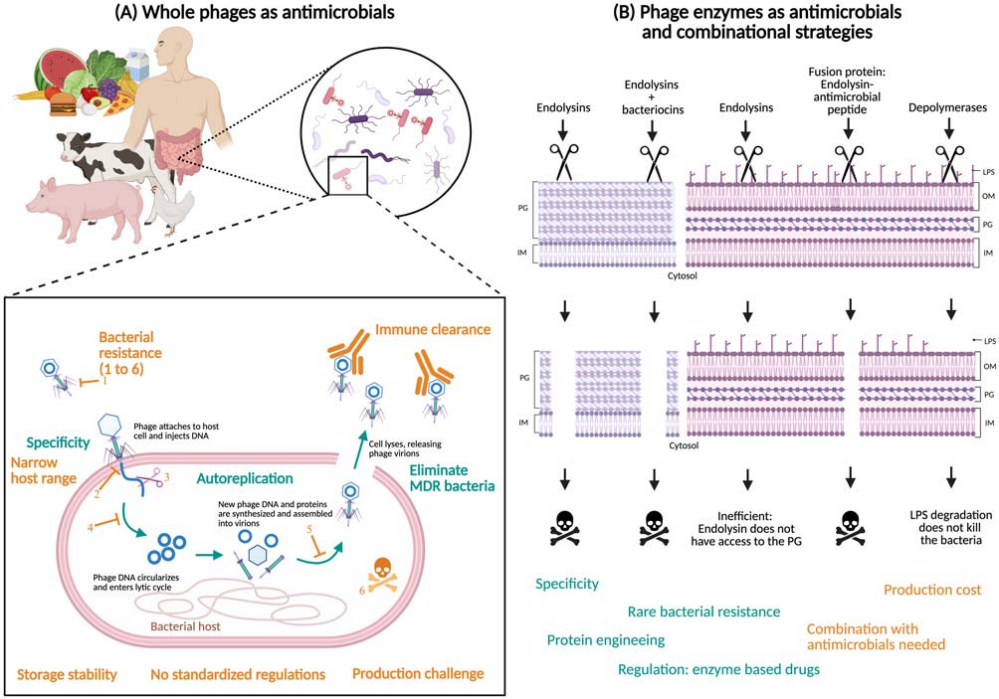
Overview of the advantages and limitations of phage-based therapies. **(A)** The application of whole bacteriophages as antimicrobial agents in human medicine, veterinary medicine, and the food industry offers several advantages (blue text) but is also associated with multiple limitations (orange text). The main mechanisms of bacterial resistance to phages are numbered from 1 to 6: inhibition of phage adsorption, blockage of DNA injection, degradation of injected phage DNA, inhibition of phage DNA replication, inhibition of phage assembly, and bacterial suicide. **(B)** These challenges may be addressed through the use of phage-derived components or combinatorial therapeutic strategies. PG, peptidoglycan; IM, inner membrane; LPS, lipopolysaccharide; OM, outer membrane. Created with BioRender.com.

Phages offer several advantages as antimicrobial agents (Figure 1). One of their most significant strengths is their high specificity: phages target only the pathogenic bacterial strains they infect, leaving the commensal microbiota unaffected. This selective action preserves the natural human gut microbiota in therapeutic applications and the microbial balance of foods in biopreservation contexts (Hu et al., 2018). In contrast to conventional preservation methods—such as pasteurization, irradiation, or chemical disinfectants—that act indiscriminately, phages represent a natural, precise, and ecologically friendly solution.

Despite their promise, phage therapy faces several limitations (Figure 1). A central challenge is the development of bacterial resistance. Bacteria can escape phage infection through various mechanisms, such as receptor modification or activation of defense systems like CRISPR-Cas (Egido et al., 2022). However, phages may coevolve in response, often at a fitness cost to the bacteria. These adaptations can reduce bacterial virulence, impair colonization, and increase sensitivity to environmental stressors or antibiotics (Azam and Tanji, 2019; Chatterjee et al., 2019; Warring et al., 2022) (Supplementary Material Table 1). Still, phage resistance remains a major concern, and understanding coevolutionary dynamics is critical for therapeutic success (Mäntynen et al., 2021).

The narrow host range of phages (it is important to note here that broad host range phages are increasingly being isolated, but even these phages do not have the ability to infect and kill a wide range of bacteria when compared to an antibiotic for example), while contributing to their specificity, also limits their broad application. Their effectiveness depends on identifying and matching the right phage to the bacterial strain, which delays treatment and restricts generalized use (Cohan et al., 2020). Moreover, their sensitivity to heat, pH, UV light, and immune clearance complicates formulation and delivery. Advances such as phage encapsulation in nanoparticles, liposomes, and hydrogels have shown promise in improving stability and bioavailability, yet optimized delivery systems are still under development (Kumari et al., 2023; Silva et al., 2023). However, it has been reported that phage formulation strategies, such as encapsulation in nanoliposomes or incorporation into films, may reduce phage viability and, consequently, their efficacy, depending on the matrix used, storage conditions, and encapsulation efficiency, which can be very low—for instance, 7.1% for mycobacteriophage D29 encapsulated in nanoliposomes (Silva et al., 2023; López de Dicastillo et al., 2021) (Supplementary Material Table 1).

Another concern is the potential for phages to mediate the horizontal transfer of antibiotic resistance genes via transduction. While some studies rigorously screen phage genomes for virulence and resistance genes, others omit this step, raising safety concerns regarding unintended gene transfer. This oversight may undermine the credibility of phage therapy and highlights the need for thorough characterization protocols (Egido et al., 2022).

Phage immunogenicity presents an additional challenge. The immune system may produce neutralizing antibodies in response to phage exposure, reducing their effectiveness, especially during repeated or prolonged treatment (Majewska et al., 2015; Stellfox et al., 2024). While some evidence suggests that anti-phage antibodies do not always hinder therapeutic success (Łusiak-Szelachowska et al., 2016), the potential for immune interference must be considered, particularly for chronic or systemic infections (Supplementary Material Table 1).

Finally, the absence of a globally standardized regulatory framework impedes the clinical integration of phage therapy. As biological entities that defy conventional drug classifications, phages face inconsistent regulatory pathways. In many countries, their use remains confined to compassionate or experimental settings. For example, Canada permits phage use under its Special Access Program (SAP), and similar limited provisions exist in Belgium, France, and Germany (Yang et al., 2023). Meanwhile, countries like Poland, Russia, and Georgia have maintained clinical phage use since the early 20th century (Yang et al., 2023). Establishing coherent and internationally harmonized regulations will be essential to advance phage therapy into mainstream clinical practice (Cui et al., 2024; Furfaro et al., 2018).

In contrast, the use of purified phage-derived components such as Tail Spike Proteins (TSPs) or endolysins could overcome these regulatory challenges. Being smaller, well-defined enzymatic molecules that behave more like conventional biologic drugs, phage components are likely to fit more easily within existing pharmaceutical frameworks, facilitating a clearer and more standardized approval process.

## 4 Alternative strategies based on phage components

In response to the growing shortage of alternatives to antibiotics and the limitations associated with the use of whole phages as antimicrobial agents, attention has turned to bacteriophage-derived proteins with antimicrobial activity. During bacterial infection, phages mobilize specific proteins capable of targeting and degrading essential bacterial structures, such as LPSs or peptidoglycan. These enzymatically active phage proteins exert their antimicrobial effect by hydrolyzing the structural polymers necessary for bacterial survival (Zhydzetski et al., 2024). The cell wall is an attractive target, as it allows the differentiation of prokaryotic from eukaryotic cells, ensuring safety, but also because of its species-specific composition, which guarantees the specificity of bacteriophage-derived proteins. Furthermore, the use of bacteriophage-derived enzymes has shown a lower emergence of bacterial resistance compared to whole phages (Loeffler et al., 2001; Schuch et al., 2002; Totté et al., 2017). As self-replicating entities, bacteriophages interact dynamically with bacteria, leading to continuous coevolution between phages and their bacterial hosts. As described previously, when a bacterium is infected by a phage, it can develop a range of resistance mechanisms, and consequently, phages develop strategies to overcome host resistance mechanisms (Azam and Tanji, 2019; Egido et al., 2022). Based on the principle of coevolution, bacteria can subsequently develop new resistance mechanisms in response to these phage adaptations, and this cycle can continue indefinitely, making phage resistance an inherent and unavoidable challenge. In contrast, the use of phage-derived endolysins or depolymerases has been associated with a low incidence of resistance (Loeffler et al., 2001; Schuch et al., 2002; Totté et al., 2017). Endolysins target the peptidoglycan, an essential component of the cell wall, that is unlikely to be modified by host bacteria since this is a highly conserved component that is essential for cell viability (Sabur et al., 2025). Depolymerases, on the other hand, degrade bacterial polysaccharide structures such as LPSs or biofilms without directly killing bacterial cells (Knecht et al., 2020). As a result, they do not exert strong selective pressure for resistance development. Since resistance emergence is one of the major causes of therapeutic failure, enzyme-based phage therapies appear more sustainable due to the rarity and low likelihood of resistance development.

### 4.1 Endolysins

Endolysins are enzymes encoded by bacteriophages that target and degrade the peptidoglycan of their specific bacterial hosts. They are promising candidates to replace conventional antibiotics due to their remarkable advantages: host specificity, mode of action, absence of toxic effects, and low risk of resistance (Pattnaik et al., 2024). These lytic enzymes have already proven to be effective in the fields of food safety, human health, and veterinary sciences (Supplementary Material Table 2). Unlike broad-spectrum antibiotics, and similarly to whole phages, endolysins specifically target certain bacterial strains without disrupting the surrounding microbiota. Combined with the absence of resistance mechanisms developed by bacteria so far, endolysins represent an increasingly studied therapeutic alternative for the treatment of MDR bacteria (Ding et al., 2020; Gonçalves et al., 2024; Guo et al., 2017; Haddad Kashani et al., 2017; Kim et al., 2020; Sabur et al., 2025; Totté et al., 2017; Vacek et al., 2023). Unlike antibiotics, which target bacterial metabolic pathways, endolysins directly attack the physical structure of the bacterial cell, the peptidoglycan. This approach makes the emergence of resistant mutants difficult, since any alteration of the peptidoglycan has a high probability of compromising cell integrity.

Endolysins are naturally used by phages to hydrolyze the bacterial cell wall from the inside and induce osmotic lysis at the end of the replication cycle (Khan et al., 2023). Purified endolysins have been shown to degrade conserved essential elements of peptidoglycan from the outside. Endolysins can be classified according to their catalytic function: N-acetylmuramoyl-L-alanine amidase, lysozyme, muramidase, or peptidase/endopeptidase (Vidová et al., 2014). They may contain multiple catalytic domains (CHAP, amidase, muramidase, chitinase, peptidase) and binding domains (CW-7, LysM, etc.), conferring adjustable efficiency and specificity depending on bacterial targets (Bocanova et al., 2022; Vidová et al., 2014; Wang et al., 2022). Endolysins have demonstrated high stability across wide ranges of pH and temperature, facilitating their use in various clinical or industrial environments (Supplementary Material Table 2). For example, LysSE24, which possesses N-acetylmuramidase activity, retains its effectiveness at pH levels from 4.0 to 10.0 and at temperatures up to 60 °C (Ding et al., 2020), while others such as EN534, Ply1228, or LysPA26 are also active under varied but narrower conditions (pH 5.0–8.0 and 20–37 °C) (Bocanova et al., 2022; Guo et al., 2017; Lai et al., 2020; Wang et al., 2022).

They can be applied exogenously as antimicrobial agents, particularly against Gram-positive bacteria, whose cell wall is not protected by an outer membrane as in Gram-negative bacteria (Ajuebor et al., 2016; Ho et al., 2021; Schmelcher et al., 2012). For example, endolysin EN534 showed activity against *B. subtilis* and *Lactobacillus jensenii*, while Ply1228 was effective against *Streptococcus suis* (Bocanova et al., 2022; Wang et al., 2022) (Supplementary Material Table 2). Similarly, the combined application of LysA and LysB resulted in visible morphological changes in *Mycobacterium smegmatis* (Lai et al., 2015). The therapeutic potential of these enzymes is supported by recent clinical trials (Buzikov et al., 2023) and experimental demonstrations of efficacy against MDR pathogens (Kim et al., 2020) (Supplementary Material Table 2). However, a study of endolysin PlyPH showed that its activity depended on the age of the target bacterial culture. PlyPH exhibited very effective activity against bacteria in the exponential phase, but its activity was reduced in the stationary phase (Paskaleva et al., 2015). These results suggest limited application under real infection conditions and highlight the need to study endolysin efficacy at all stages of bacterial development to determine their prophylactic or curative potential.

The effectiveness of phage endolysins is not limited to clinical contexts: industrial applications have also been explored. Endolysins have demonstrated utility in preventing and eradicating bacterial contaminants in the food industry and processing facilities (Suja and Gummadi, 2024). They have been used to reduce bacterial load on food production surfaces or in food matrices and incorporated into antimicrobial packaging or disinfection treatments targeting pathogens such as *L. monocytogenes, S. aureus, Salmonella, E. coli, C. jejuni, B. cereus*, or *C. perfringens* (Grigore-Gurgu et al., 2024; Nazir et al., 2023; Suja and Gummadi, 2024) (Supplementary Material Table 2).

However, the exogenous use of endolysins as antimicrobials against Gram-negative bacteria remains limited, due to their outer membrane, which acts as an impermeable barrier preventing endolysins from directly accessing the peptidoglycan. This limitation has been partially overcome through various strategies, including combination with outer membrane-permeabilizing agents, protein engineering to create endolysins containing domains that facilitate passage through the outer membrane, fusion of endolysins with antimicrobial peptides to disrupt the outer membrane and allow the protein to reach its target, or encapsulation in delivery systems such as liposomes, which enhances endolysin transport and delivery to target peptidoglycan (Abdelrahman et al., 2021; Gonçalves et al., 2024; Lai et al., 2020; Schmelcher et al., 2012; Sisson et al., 2024) (Supplementary Material Tables 2, 3). These approaches expand the spectrum of endolysin activity to Gram-negative bacteria. Nevertheless, although endolysins are naturally active against Gram-positive bacteria, with statistically comparable effectiveness to phages (Ssekatawa et al., 2021), chimeric endolysins have already been developed with the aim of improving their stability, solubility, and lytic efficiency (Choi et al., 2023; Haddad Kashani et al., 2017; Vacek et al., 2023).

### 4.2 Depolymerases

Polysaccharide depolymerases are enzymes encoded by bacteriophage genomes that degrade carbohydrate structures present on the surface of bacteria, such as capsules, LPSs, and biofilm matrices (Latka et al., 2017; Pires et al., 2016). These enzymes, as integral part of TSPs, facilitate the access of phages to their bacterial receptors by locally degrading the polysaccharide barrier that protects the cell surface. A comparative genomic analysis identified 160 putative depolymerases in 143 phage genomes infecting 24 bacterial genera (Pires et al., 2016). They are commonly classified as hydrolases (e.g., sialidases, levanases, dextranases) or lyases (e.g., hyaluronidases, pectate lyases, alginate lyases), each targeting specific glycosidic bonds on the bacterial surface (Pires et al., 2016). Structurally, most depolymerases adopt elongated trimeric β-helical architectures, such as the well-characterized TSP of phage P22, and exhibit resistance to protease, SDS and heat (Chen and King, 1991; Seul et al., 2014; Steinbacher et al., 1997). Their modular organization typically includes an N-terminal domain (also known as dome-like structure) anchoring the enzyme to the phage head, and a C-terminal domain involved in receptor recognition and binding and possessing the endoglycosidase activity that degrades the O-antigen polysaccharide (Steinbacher et al., 1997). These two domains are connected by a short and flexible linker peptide, facilitating the attachment of the TSPs to the receptors (Seul et al., 2014). Their specificity to distinct capsular types or O-antigen motifs often defines the host range of their corresponding phages (Knecht et al., 2020) (Supplementary Material Table 1).

Depolymerases act through specific hydrolytic or eliminative cleavage of glycosidic linkages, producing soluble oligosaccharides and exposing the bacterial membrane for genome injection (Seul et al., 2014). Phage TSPs possess carbohydrate depolymerase activity and recognize and cleave components of the LPS to position the phage toward a secondary membrane receptor during infection (Andres et al., 2012). TSPs are highly thermostable and protease resistant (Ayariga et al., 2021) and have also been observed to decrease bacterial viability (Supplementary Material Table 2). For example, Ayariga et al. (2021) recently demonstrated that the ε34 phage tailspike protein has enzymatic property as a LPS hydrolase, and synergizes with Vero Cell culture supernatant in killing *S*. Newington. Earlier, Miletic et al. (2016) expressed the receptor binding domain of the phage P22 Gp9 tailspike protein in plant tissue (*Nicotiana benthamiana*), and demonstrated that, upon oral administration of lyophilized leaves expressing Gp9 TSP to newly hatched chickens, *Salmonella* concentrations were reduced on average by approximately 0.75 log relative to controls. Others have shown that TSPs can be used to control the growth of plant pathogens. For example, expression of the *Erwinia* spp. phage TSP DpoEa1h in transgenic apple and pear plants significantly reduced fire blight (*Erwinia amylovora*) susceptibility (Malnoy et al., 2005), likely due to removal of the main virulence factor amylovoran and exposing the *E. amylovora* cells to host plant defenses (Kim et al., 2004). Collectively, these studies demonstrate the utility of TSPs as novel antimicrobials to control the growth of food and plant-borne pathogens in foods.

Beyond their role in infection, their enzymatic activity directly disrupts biofilm matrices, disaggregating bacterial communities and increasing their susceptibility to antimicrobials and host immune responses, and decreasing the virulence of pathogenic bacteria (Lin et al., 2018; Wang et al., 2024). Their anti-virulence mechanism has been demonstrated against numerous MDR pathogens, including *K. pneumoniae, P. aeruginosa, A. baumannii*, and *E. coli* (Knecht et al., 2020; Latka et al., 2017; Wang et al., 2024) (Supplementary Material Table 2). In animal models, depolymerase treatment significantly reduced bacterial load and improved survival, underscoring their therapeutic promise (Wang et al., 2024). Unlike whole phages, purified depolymerases do not replicate or transfer genetic material, which enhances their biosafety profile and regulatory acceptability.

Despite their potential, several limitations hinder their clinical translation. Their high substrate specificity, while advantageous for precision targeting, restricts their applicability to a narrow range of bacterial strains. Furthermore, although rare, resistance can emerge through mutations or structural modifications of surface polysaccharides, though such adaptations often reduce bacterial virulence (Wang et al., 2024). From a technological standpoint, depolymerase formulation remains a major challenge (Wang et al., 2024). Most studies have been limited to liquid preparations, which severely restrict stability and shelf life, even after optimization, typically up to 6 months without significant loss of activity (Wang et al., 2024). Immunogenicity assessment of a depolymerase targeting *K. pneumoniae* revealed that, although it elicited an immune response and the production of neutralizing antibodies, its antimicrobial activity remained unaffected. However, such evaluations should be expanded to a broader range of depolymerases (Bansal et al., 2014). Many depolymerases have been studied in preclinical phases, and available data suggest that with appropriate dosing regimens, they are capable of effectively and safely combating various MDR pathogens in animal infection models. However, treatment delays significantly reduce the effectiveness of these enzymes (Lin et al., 2018).

Recent research has also focused on the engineering and modular recombination of depolymerases to broaden their host range or improve catalytic efficiency (Dunne et al., 2021; Latka et al., 2021) (Supplementary Material Table 2). Domain swapping, directed evolution, and computational design are used to modify substrate-binding regions and enhance stability under physiological conditions (Latka et al., 2021). Overall, polysaccharide depolymerases represent a powerful class of enzymatic tools that combine precision, stability, and anti-virulence activity. Although their development is still at a preclinical stage, overcoming current challenges related to formulation, production, and host specificity could unlock their therapeutic and industrial potential as alternatives or complements to conventional antimicrobials in the treatment of infections caused by MDR bacteria.

### 4.3 Advantages and disadvantages of phage components

Despite the promising potential of bacteriophage-derived proteins as antimicrobial agents, several obstacles hinder their large-scale development and clinical application (Figure 1). Among them, technological challenges associated with the industrial production of these therapeutic proteins remain major, particularly regarding their yield, stability, and purification costs.

Large-scale production of phage enzymes for clinical use poses significant challenges. These proteins are produced via recombinant expression in prokaryotic systems, where production must be optimized for each enzyme. They must then undergo rigorous purification steps—ultrafiltration, chromatography, ultracentrifugation—to remove all bacterial contaminants, which are complex and costly processes. A molecule intended for medical use must be prepared according to Good Manufacturing Practices (GMP), which are the standard for ensuring drug quality, safety, and efficacy (Rivera-Lopez et al., 2025). It must contain no proteins or DNA from the host cell in which it was expressed, nor any exotoxins or endotoxins. Indeed, the final administered product must achieve extreme purity to avoid any immunogenicity (Cooper et al., 2016). Developing an efficient expression and purification system is essential to reduce production costs and the *in vivo* toxicity of the molecule (Cooper et al., 2016; Choi et al., 2025). Similarly, to produce whole phages (or phage cocktails), preparations must be produced following the same GMP guidelines to ensure an ultrapure final product (Cooper et al., 2016). Scaling up phage production represents a technological challenge, particularly in the choice of host bacteria. The host must be non-pathogenic, genetically stable, and incapable of producing harmful byproducts (Wójcicki et al., 2025). However, due to the narrow host range and specificity of a phage, it may sometimes be impossible to meet these criteria, as the only possible host is a pathogen. Finally, a phage preparation (either a single phage or a cocktail) must be titrated (plaque-forming units (PFU) for each phage) and characterized (storage stability and purity) (Wójcicki et al., 2025). Thus, although phage enzyme production is costly, it benefits from a “classic” recombinant protein production process that is predictable, whereas phage preparations face technological challenges related to host selection and standardization of composition, making them less predictable in terms of efficacy.

Furthermore, the absence of a clear and harmonized regulatory framework governing their use represents a considerable barrier to their market entry and acceptance by health authorities. However, given that enzymes are already used and considered safe in food and feed ingredients, the therapeutic application of phage-derived enzymes could follow similar regulatory and safety pathways.

Whole bacteriophages are subject to complex regulatory frameworks due to their dynamic, self-replicating biological nature and the presence of viral genetic material, which distinguishes them from conventional antimicrobial products (Rivera-Lopez et al., 2025). Their regulatory classification strongly depends on the intended application, as bacteriophages may be regulated as biological products, food additives, or biopesticides depending on the jurisdiction and use scenario (Rivera-Lopez et al., 2025; FDA, 2021). In the United States, therapeutic applications of whole bacteriophages fall under the authority of the Food and Drug Administration (FDA), primarily through the Center for Biologics Evaluation and Research (CBER), while agricultural and environmental applications may involve the Environmental Protection Agency (EPA), and food-related uses may be overseen by the FDA and USDA/FSIS (FDA, 2021; Rivera-Lopez et al., 2025). In Europe, the absence of a harmonized regulatory framework for whole phages results in case-by-case evaluations by the European Medicines Agency (EMA) or the European Food Safety Authority (EFSA), depending on whether the intended use is therapeutic or food-related (Choi et al., 2025). Concerns related to *in situ* replication, batch-to-batch variability, the frequent use of phage cocktails, and the theoretical risk of horizontal gene transfer contribute to challenges in standardization, quality control, and risk assessment, thereby slowing regulatory approval for whole bacteriophages (Niazi, 2025).

In contrast, phage-derived enzymes and phage-based compounds, such as endolysins and depolymerases, lack genetic material and replicative capacity and are therefore more readily aligned with existing regulatory frameworks (Cooper et al., 2016). These molecules are generally classified as antimicrobial enzymes or conventional biologics, categories for which regulatory pathways are already well established, particularly in the food and biotechnology sectors (Cooper et al., 2016; Choi et al., 2025). Their defined molecular composition, structural stability, and compatibility with Good Manufacturing Practice (GMP) requirements enable more consistent and simplified safety evaluations, including GRAS (Generally Recognized as Safe) determinations, food enzyme assessments, or food contact substance notifications in the United States (Cooper et al., 2016; FDA, 2021; Rivera-Lopez et al., 2025). Consequently, phage-derived antimicrobial enzymes follow simpler and faster regulatory trajectories than whole bacteriophages, making them particularly attractive alternatives for both agri-food and biomedical applications.

Moreover, to maximize their *in vitro* and *in vivo* effectiveness, these proteins often need to be combined with permeabilizing agents that facilitate access to bacterial targets or encapsulated in delivery systems that allow targeted and controlled release. Nevertheless, these biomolecules present several notable advantages: their high specificity limits disturbances to the commensal microbiota, their enzymatic mode of action makes the emergence of resistance more difficult, and their low molecular weight facilitates engineering and formulation. The studies include in this review rely primarily on *in vitro* approaches or simplified experimental models that do not fully capture the complexity of *in vivo* conditions, thereby limiting the extrapolation of the results to real clinical or environmental settings (Ayariga et al., 2021) (Supplementary Material Table 2). Several studies exhibit restricted or insufficiently controlled experimental designs, small sample sizes, or specific conditions in terms of dosage, timing, or pretreatment, which complicates data interpretation and limits generalizability (Bansal et al., 2014; Ding et al., 2020; Kim et al., 2004; Lin et al., 2018; Malnoy et al., 2005; Totté et al., 2017). The high bacterial specificity of endolysins and depolymerases, while advantageous for targeted applications, also represents a major limitation for broad-spectrum use (Gonçalves et al., 2024). In addition, the molecular mechanisms of action and the structural determinants underlying enzymatic efficacy often remain only partially elucidated, highlighting the need for further investigation (Guo et al., 2017; Haddad Kashani et al., 2017). Finally, multiple studies point to engineering-related constraints, including issues of enzyme stability and folding, as well as a lack of sufficient data on long-term efficacy or performance under realistic conditions, which hampers translation toward therapeutic or agricultural applications (Choi et al., 2023; Latka et al., 2021; Paskaleva et al., 2015; Seul et al., 2014). Thus, although limitations remain, phage-derived compounds provide a solid foundation for the development of innovative and targeted antimicrobial therapies (Supplementary Material Table 2).

## 5 Combinational strategies

In response to the difficulties in treating diseases caused by antibiotic resistant bacteria, numerous strategies have been proposed to improve the effectiveness of therapies based on the use of bacteriophages or phage-derived compounds. As summarized in the review by Suja and Gummadi (2024), approaches such as the use of multi-phage cocktails and combinations with conventional antimicrobial agents have been explored to broaden the activity spectrum of phages and bypass bacterial barriers. These approaches enhance the bactericidal effectiveness of each compound used by overcoming the limitations inherent to each strategy, such as penetration into eukaryotic cells to reach intracellular pathogens, or improving the activity of phage proteins or antimicrobial agents that are ineffective against Gram-negative bacteria due to the structure of their outer membrane (Supplementary Material Table 3). Another notable advantage of these synergistic associations is the significant reduction in the quantity of combined compounds required, and consequently, the reduction of side effects and toxicity. These combinations offer new perspectives for the development of antimicrobial therapies that are more effective and more targeted.

### 5.1 Endolysins combined with bacteriocins

Phage lytic proteins can also be combined with bacteriocins to enhance antibacterial potency. Previous studies have shown that a bacterial bacteriocin such as lysostaphin (from *S. simulans*) acts synergistically with endolysin LysK from phage K to kill methicillin-resistant *S. aureus* (MRSA). Endolysin LysK, possessing two catalytic domains, potentiates the lytic effect of lysostaphin, which has only one (Mathur et al., 2017). Similarly, nisin can amplify the activity of phage endolysins: when combined with endolysin LysH5 specific for *S. aureus*, nisin enhanced staphylococcal destruction by permeabilizing the cell wall, thereby facilitating enzyme access to its target (Mathur et al., 2017). These enzyme–bacteriocin duos take advantage of complementary modes of action (cell wall degradation by the enzyme, membrane perforation or biosynthesis inhibition by the bacteriocin). Once again, the benefit is targeting the bacterium on multiple fronts simultaneously to prevent the emergence of escape variants.

### 5.2 Fusion proteins: endolysins–antimicrobial peptides

A recent strategy to attack MDR bacteria, especially Gram-negative ones, is to fuse a phage protein with an antimicrobial peptide. The added peptide, often cationic, helps the enzyme cross the outer membrane of Gram-negative bacteria and reach the peptidoglycan. These constructs have proven to be very powerful. For example, the endolysin PA90 from a *Pseudomonas* phage was fused with thanatin (an insect antimicrobial peptide): the resulting product, named Tha-PA90, exhibited bactericidal activity far superior to that of thanatin alone, including against MDR *A. baumannii* strains (Lim et al., 2024). *In vitro*, Tha-PA90 rapidly killed these bacteria while being non-toxic to human cells, and *in vivo* it significantly improved the survival of infected mice, whereas the endolysin alone initially had poor activity (Lim et al., 2024). Similarly, the company Artilysation developed the artilysin Art-175 (a fusion of an endolysin with an amphiphilic peptide): this fusion protein demonstrated efficient killing of MDR *P. aeruginosa* and even persistent biofilm forms, where the native endolysin and peptides alone were ineffective (Sabur et al., 2025). These hybrid proteins therefore broaden the action spectrum of phage enzymes and represent a promising avenue for targeting highly resistant pathogens (e.g., *A. baumannii, P. aeruginosa*, and even MRSA). It should be noted that these approaches are still under preclinical development, and further studies will be needed to assess their immunological safety and large-scale *in vivo* efficacy as well as their acceptance as genetically-modified variants.

### 5.3 Phage enzymes and essential oils

Essential oil compounds possess antibacterial properties (membrane permeabilization, efflux pump inhibition, cell wall weakening) that can complement those of phages. For example, the phage endolysin LysSA97 combined with carvacrol (a major aromatic phenol from thyme essential oil) exhibited a synergistic bactericidal effect against *S. aureus*: in contaminated skim milk, this duo reduced the staphylococcal population below the detection threshold, whereas neither agent alone achieved this (Soto Lopez et al., 2025). The carvacrol makes the *S. aureus* membrane more permeable, thereby enhancing the action of the endolysin on the peptidoglycan (Soto Lopez et al., 2025). Conversely, complex matrices can attenuate this synergy: in whole milk (rich in lipids), the same LysSA97–carvacrol combination showed no superior effect, likely because fats hinder the access of the carvacrol to the bacteria (Soto Lopez et al., 2025). This suggests that essential oils can increase bacterial sensitivity to phages or enzymes (by disrupting physical barriers or defense systems) (Fokas et al., 2025), but their effectiveness depends on the medium and the bioavailability of the active compound. Similar studies with other oils (cinnamon or oregano) and phages sometimes indicate variable results, highlighting the importance of adapting the combination to the context of use (food, clinical, biofilm, for example).

## 6 Conclusion

In summary, combinations of phages or phage-derived proteins with antimicrobial agents offer original and powerful strategies to tackle MDR bacteria (Figure 1). By exploiting their complementary mechanisms of action, these combinations achieve higher bacterial clearance rates, biofilm eradication, and reduced probability of the emergence of resistance against the combined agents (Mathur et al., 2017; Szymczak et al., 2024). However, some combinations exhibit reduced efficacy compared with conventional antibiotics, demonstrating the need for further optimization of the combination to enhance their efficacy (Lim et al., 2024). Although challenges remain, such as the standardization of optimal doses, the risk of antagonism at high concentrations, or regulatory considerations for the approval of such dual approaches, the literature since 2015 highlights numerous conclusive applications. These findings suggest that combinatorial therapies involving phages, their enzymes, or other biomolecules could become tools of choice to treat diseases caused by MDR pathogenic bacteria in the light of the increasingly failure of conventional approaches.
