# Supplementary material for: Phages as antimicrobials against multi-drug resistant bacteria
Source: Front Microbiol. 2026 Feb 23;17:1747240. doi: 10.3389/fmicb.2026.1747240 (PMC12968171; doi:10.3389/fmicb.2026.1747240)
Supplement: Supplementary file 1 [file Data_Sheet_1.pdf]

Supplementary Material

1 Supplementary Tables

Supplementary Table 1. Overview of experimental studies and reviews on the use of whole bacteriophages as antimicrobial agents included in this narrative review.

Supplementary Table 2. Overview of experimental studies and reviews focusing on endolysins and depolymerases included in this narrative review.

Supplementary Table 3. Overview of experimental studies and reviews addressing combinatorial phage-based strategies included in this narrative review.
